# Impact of phototherapy on gut microbiota composition and function in neonates with hyperbilirubinemia: a metagenomic analysis

**DOI:** 10.1186/s12887-026-06531-0

**Published:** 2026-02-02

**Authors:** Mengjie Luo, Xiaodong Xiao, Yi Wu

**Affiliations:** 1 Department of Clinical Laboratory Science, Shenzhen Yantian District People’s Hospital, 2010 Wutong Road, Yantian District, Shenzhen, 518081 China; 2 Department of Pediatrics, Shenzhen Yantian District People’s Hospital, Shenzhen, 518081 China

**Keywords:** Neonatal hyperbilirubinemia (NH), Phototherapy, Gut microbiome, High-throughput sequencing, Metagenomics

## Abstract

**Background:**

Phototherapy serves as the primary treatment for neonatal hyperbilirubinemia (NH). This research aims to investigate the impact of phototherapy on the gut microbiota of NH, and to provide reliable theoretical evidence for the clinical application of phototherapy in such cases.

**Methods:**

In this self-controlled longitudinal study, 26 newborns diagnosed with NH were enrolled. Fecal samples were collected before (pre-treatment) and 48 h after (post-treatment) initiating phototherapy. The gut microbiota was profiled using high-throughput 16 S ribosomal RNA (rRNA) gene sequencing. Gut microbiota composition and diversity were analyzed using standard bioinformatics pipelines. Data were processed with standard bioinformatics tools for taxonomic annotation, diversity analysis, and functional prediction based on the COG, KEGG, and MetaCyc databases. Statistical significance was assessed using the Wilcoxon signed-rank test (*P* < 0.05).

**Results:**

While no significant differences were observed at the species level, analysis at the genus level revealed significant alterations in the gut microbiota. The genera *Clostridium* and *Megamonas* were identified as significantly increased post-phototherapy. Linear discriminant analysis effect size (LEfSe) analysis further confirmed distinct microbial signatures between the two groups: pre-treatment samples were enriched with families such as Porphyromonadaceae, Lachnospiraceae, Alcaligenaceae, Ruminococcaceae, Moraxellaceae, and the order Pseudomonadales. In contrast, post-treatment samples were predominantly characterized by the class Erysipelotrichi and its associated taxa (Erysipelotrichales and Erysipelotrichaceae). α-diversity indices (Sobs, Chao, Shannon, Simpson) showed no significant differences between the two groups, whereas β-diversity analysis indicated significant microbial community separation (*P* < 0.05). Predicted functional profiles (based on 16 S rRNA gene data using PICRUSt2) suggested predominant roles in metabolism, genetic information processing, and biosynthesis. However, no significant differences were observed between the pre- and post-treatment groups.

**Conclusions:**

Phototherapy significantly modulated the gut microbial composition of neonates with NH, notably increasing the abundance of *Clostridium* and *Megamonas*, and shifting the community towards Erysipelotrichi, while overall microbial functional capacity remained stable. These findings highlight the dynamic yet resilient nature of the neonatal gut microbiota under phototherapy and provide a foundation for microbiome-informed management strategies in neonatal hyperbilirubinemia.

**Supplementary Information:**

The online version contains supplementary material available at 10.1186/s12887-026-06531-0.

## Introduction

**Fig. 1:**
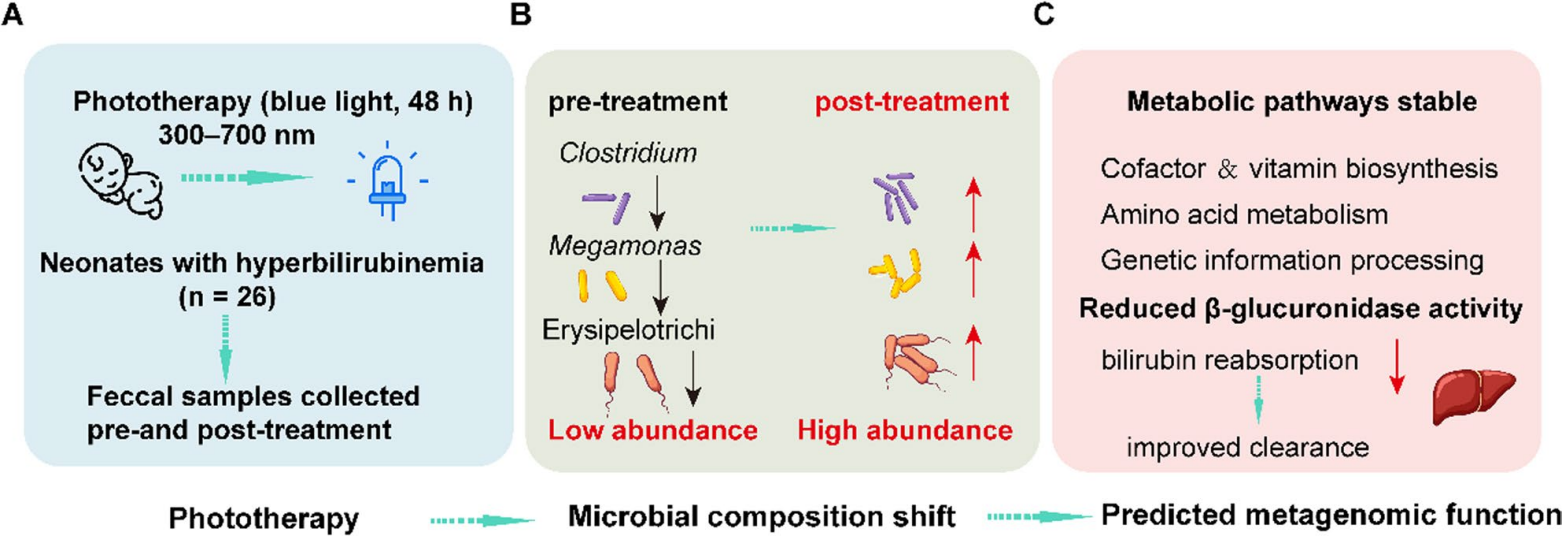
Proposed mechanistic model of phototherapy-induced modulation of gut microbiota in neonatal hyperbilirubinemia. **A** Phototherapy. **B** The related alterations in the relative abundance of specific bacterial taxa. **C** A functional resilience of the neonatal gut ecosystem under phototherapy exposure (COG/KEGG/MetaCyc)

Neonatal hyperbilirubinemia (NH) is a common clinical condition characterized by elevated bilirubin levels, affecting newborns during the neonatal period. The condition can be divided into physiological and pathological jaundice, with the latter posing serious risks, including acute bilirubin encephalopathy and kernicterus, if not properly managed [1, 2]. Phototherapy is widely recognized as the first-line treatment for NH due to its non-invasive nature and proven efficacy in leveraging blue light (450–470 nm) to convert serum bilirubin into water-soluble derivatives, thereby facilitating its excretion via urine and stool [3, 4]. It also decreases the need for an exchange transfusion. Despite the established clinical benefits of phototherapy, emerging concerns have been raised regarding its potential for short and long-term adverse effects. Recent studies suggest that light exposure may influence various physiological systems, including circadian regulation, immune responses, thermoregulatory imbalance, diarrhea, sleep disturbances, and the development of bronze-like skin discoloration [5, 6]. Critically, preliminary research proposes that some side effects may stem from phototherapy-induced gut microbiota disturbance [7]. The gut microbiota plays a vital role in metabolic programming, immune system development, and resistance to pathogens [8, 9]. It has been recognized that the gut microbiota and hepatointestinal signaling pathways are established regulators of bilirubin metabolism and elimination [10, 11]. For instance, *Lactobacillus rhamnosus* supplementation has been shown to mitigate phototherapy-induced dysbiosis and accelerate bilirubin clearance in jaundiced neonates [12]. However, the previous view was incomplete and contradictory with many phenomena due to the limited knowledge of the neonatal gut microbiota and the impact of phototherapy on neonatal gut microbiota. More evidence detailing characterization of compositional shifts at high taxonomic resolution and associated functional consequences is needed. Therefore, this study aims to investigate the structural and functional alterations in the gut microbiota of NH neonates before and after phototherapy using 16 S rRNA sequencing and bioinformatic prediction. Collectively, the changes in gut microbiota composition induced by phototherapy, as summarized in our proposed model (Fig. 1), suggest a plausible microbial component to its therapeutic mechanism. The significant increase in *Clostridium* and *Megamonas*, coupled with a distinct community shift towards Erysipelotrichi (Fig. 1B), occurred alongside stable predicted metagenomic functions. We hypothesize that this specific restructuring may contribute to bilirubin clearance, potentially through a reduction in bacterial β-glucuronidase activity, thereby decreasing intestinal bilirubin reabsorption (Fig. 1C). Our findings might provide mechanistic insights into potential side effects and supporting strategies for microbiota-targeted adjuvant therapies.

## Materials and methods

### Study subjects

This study adopted a self-controlled design, in which each neonate served as their own control. Fecal samples collected before phototherapy were defined as the pre-treatment group, and samples collected 48 h after phototherapy were defined as the post-treatment group. Twenty-six neonates diagnosed with NH and undergoing phototherapy in the Department of Neonatology, Shenzhen Yantian District People’s Hospital, between June 2021 and December 2022 were enrolled. The inclusion criteria were as follows: (1) The diagnostic and phototherapy indications for NH complied with the guidelines established by the American Academy of Pediatrics [3]; (2) Pregnancy > 35 weeks, and age ranged from 3 to 28 days; (3) Vaginal delivery and birth weight ≥ 2.5 kg. (4) Breastfeeding after birth; (5) Normal stool routine test results and absence of clinical diarrhea symptoms before phototherapy. Exclusion criteria were as follows: (1) Previous use of antibiotics, probiotics, prebiotics, or synbiotics; (2) maternal GBS infection, intrapartum fever, chorioamnionitis; (3) gastrointestinal diseases or diabetes mellitus; (4) neonatal infection, viral hepatitis, asphyxia, acidosis, or hemolytic disease. To minimize potential confounding effects on the gut microbiota, all infants were treated in the same hospital unit, standardizing the hospital environment to some extent. The study protocol was approved by the Ethics Committee of Shenzhen Yantian District People’s Hospital and the Health Commission of Shenzhen Yantian District (No. YTWS20210102). Written informed consent was obtained from the parents and/or legal guardians of the enrolled children.

### Fecal samples and general information collection

All infants received double-sided phototherapy with the same type of blue light source (spectral range 300–700 nm) and the same treatment session duration (12 h). The light source-to-infant distance was maintained within a standardized clinical range (25–35 cm). A standardized phototherapy session lasted for 12 h. Fecal samples were collected by trained nurses using sterile disposable collection tubes within 30 min before initiation of phototherapy (0 h) and at 48 h after completion. Approximately 1–2 g of freshly passed stool was immediately transferred into sterile 5 mL cryogenic tubes. All samples were flash-frozen in liquid nitrogen within 30 min of collection and then stored at − 80 °C until DNA extraction.

### Microbial DNA extraction and library construction

Microbial genomic DNA was extracted from approximately 100−200 mg of each fecal sample using the MagPure Stool DNA KF Kit B (MAGEN, China) according to the manufacturer’s instructions. Briefly, samples were homogenized with grinding beads in Buffer ATL/PVP−10 using a grinding machine (Shanghai Jingxin Tech, China), followed by incubation at 65℃. After centrifugation, the supernatant was subjected to PCI buffer treatment for purification. Subsequent DNA binding, washing, and elution steps were performed automatically using magnetic beads on a Kingfisher instrument (Thermo Fisher, USA). The purified DNA was eluted in 100 µL of elution buffer and stored at -80℃ for downstream analysis. The concentration of the extracted DNA was accurately quantified using fluorescence-based methods (Qubit Fluorometer with the Qubit dsDNA HS Assay Kit, and a microplate reader with a broad-range DNA fluorescent dye). DNA integrity was assessed by 1% agarose gel electrophoresis. Genomic DNA was subjected to rigorous quality control prior to library construction. Samples were deemed “qualified” only if they met all of the following criteria: a concentration ≥ 12.5 ng/µL, integrity demonstrated by a dominant electrophoretic band > 20 kb, and purity sufficient to rule out significant contamination from proteins, RNA, or salt ions. Only qualified DNA samples proceeded to the subsequent library preparation steps. For each qualified genomic DNA sample, 30 ng was used as template for PCR amplification with fusion primers targeting the 16 S rRNA gene. PCR products were purified using Agencourt AMPure XP magnetic beads (Beckman Coulter, USA) and eluted in Elution Buffer. The constructed libraries were evaluated using an Agilent 2100 Bioanalyzer (Agilent Technologies, USA) to confirm the expected fragment size distribution and to ensure the absence of adapter dimers or other construction artifacts. Libraries with a fragment size distribution of 300–600 bp, a concentration ≥ 5 ng/µL, a volume of 40 µL, and adapter dimer contamination below 1%, as determined by the Agilent 2100 Bioanalyzer (Agilent Technologies, USA), were considered qualified for sequencing on the Illumina HiSeq2500 platform.

### Bioinformatics analysis

Raw sequencing data were filtered to obtain high-quality clean reads for downstream analysis. FLASH (v1.2.11) was used to assemble reads into Tags based on overlap. USEARCH (v7.0.1090) clustered Tags into Operational Taxonomic Units (OTUs) at 97% similarity. Chimeras were removed using the gold database (v20110519) for 16 S or UNITE (v20140703) for Internal Transcribed Spacer (ITS). Taxonomic annotation was performed against the Greengene_16S_Chloroplast database. Species composition and diversity analyses were conducted based on OTUs and annotation results. PICRUSt2 (v2.3.0-b) was used for functional prediction against COG, KEGG, and MetaCyc databases. Alpha diversity was assessed using Sobs, Chao, ace, Shannon, and Simpson indices.

### Statistical analysis

SPSS software (version 26.0; IBM Corp.) was used to analyze the data, which are presented as the mean ± SE. The Wilcoxon test was utilized for comparisons between groups with a non-normal distribution. Correlations between variables were evaluated using the Spearman coefficient. A paired t‑test was used if the data conformed to a normal distribution. *P* < 0.05 was considered to indicate a statistically significant difference.

## Results

### Diversity analysis of gut microbiota

**Fig. 2:**
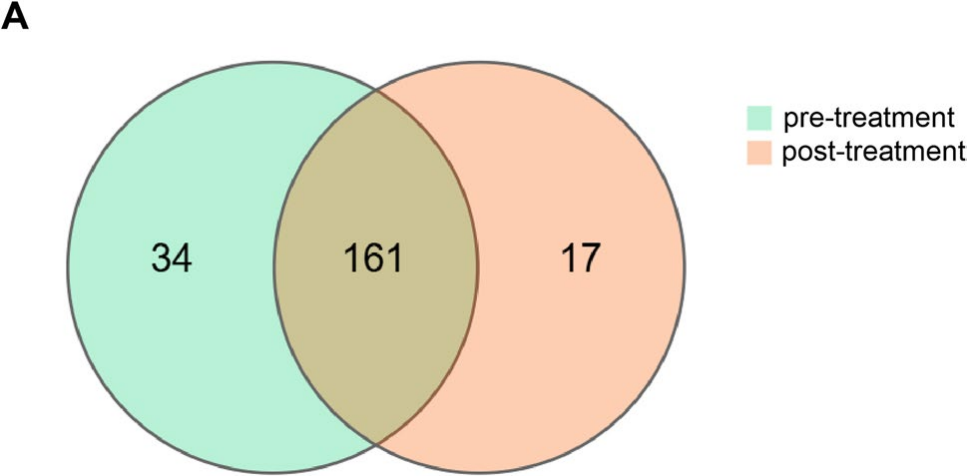
Comparison of OTUs between pre-treatment (before phototherapy) and post-treatment (after phototherapy) samples. The number in the overlapping part is the number of OTUs shared between the two groups

**Fig. 3:**
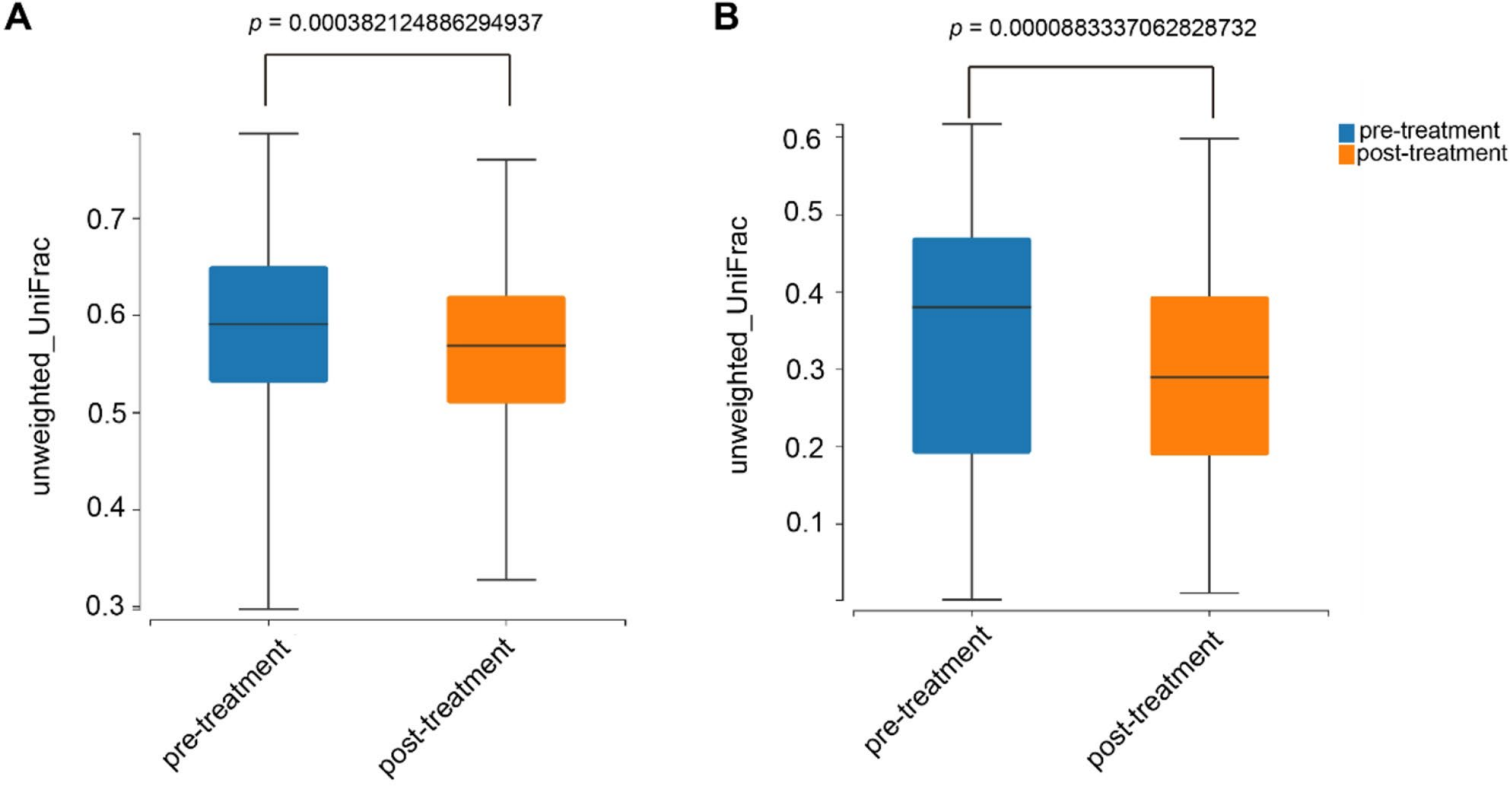
β-diversity analysis of gut microbiota before and after phototherapy. Principal Coordinate Analysis (PCoA) based on unweighted UniFrac (**A**) and weighted UniFrac (**B**) distances demonstrates distinct microbial community structures between pre-treatment (before phototherapy) and post-treatment (after phototherapy) groups. Each point represents an individual sample, and the distance between points reflects compositional dissimilarity. Statistical significance was evaluated using PERMANOVA (999 permutations), with p-values displayed on the plots. Both distance metrics indicate significant differences in overall community composition following phototherapy, suggesting a shift in gut microbiota structure independent of α-diversity changes. PERMANOVA: unweighted UniFrac, *P* = 0.000382124886294937; weighted UniFrac, *P* = 0.0000883337062828732

To evaluate the alteration of intestinal microbiota before (pre-treatment group) and after phototherapy (post-treatment group), 16 S rRNA sequencing analysis was employed on fecal samples from both groups. The species accumulation curve approached an asymptotic plateau after sequencing all 52 samples (26 paired pre- and post-phototherapy specimens), indicating sufficient sampling depth to capture the majority of microbial diversity in the neonatal gut microbiota (Supplementary Fig. S1). The Venn diagram showed that a total of 212 OTUs were identified, with 178 in the post-treatment group and 195 in the pre-treatment group, of which 161 were shared (Fig. 2). α diversity analysis revealed: Sobs (*p* = 0.27983), Chao (*p* = 0.05939), ACE (*p* = 0.01162), Shannon (*p* = 0.2328), Simpson (*p* = 0.24007), and Good’s coverage (*p* = 0.00303). Although ACE and Good’s coverage showed statistical differences, these changes were not reproduced across other α-diversity metrics, indicating no consistent alteration in gut microbial diversity following phototherapy (Supplementary Fig. S2). β diversity analysis, as shown in Fig. 3A and B, revealed a significant difference in microbial communities between the two groups based on unweighted and weighted unifrac distance (both *P* < 0.001), indicating a significant change over the phototherapy.

### Taxonomic composition analysis of intestinal microbiota abundance

**Fig. 4:**
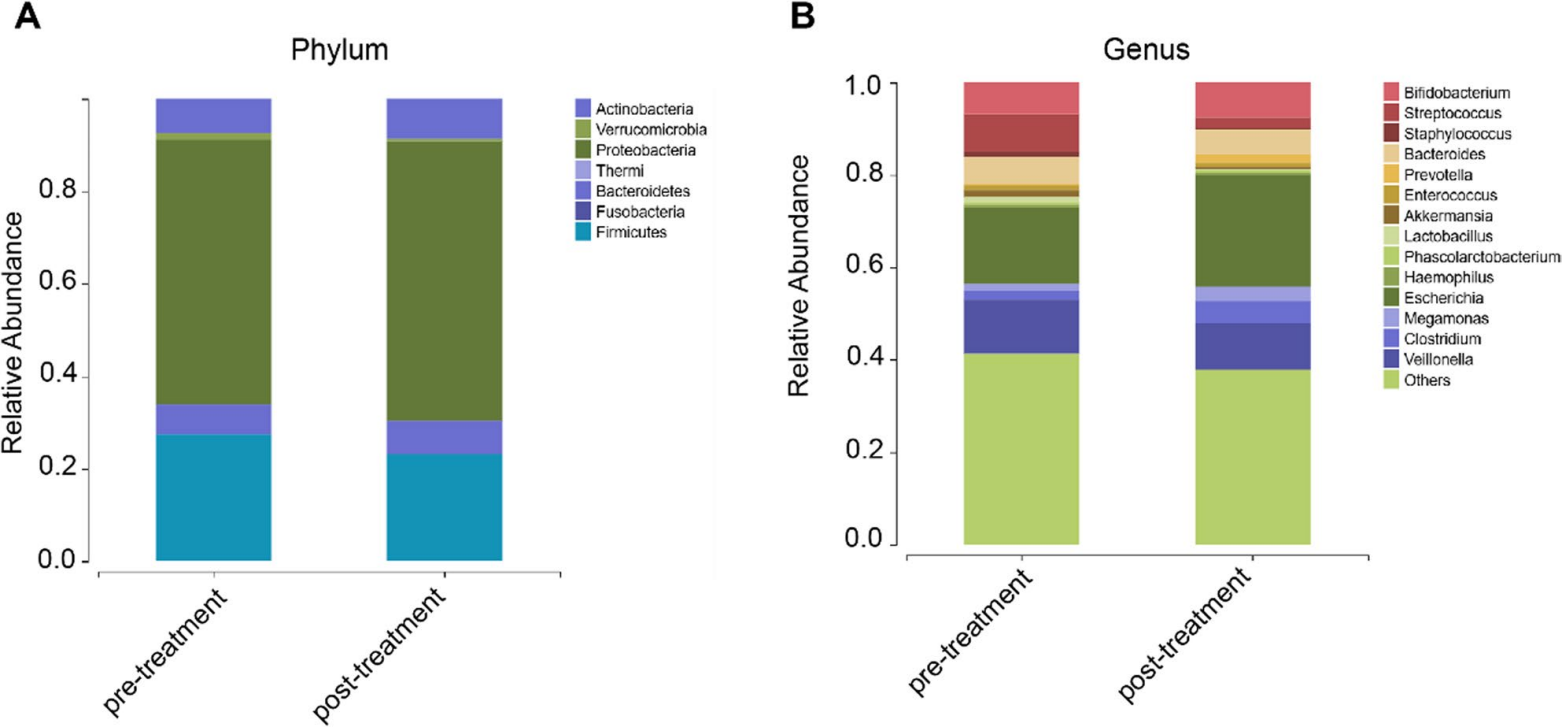
Bacterial composition of gut microbiota in NH in the pre-treatment group and post-treatment group. **A** Distribution of the predominant bacteria at the phylum level. **B** Distribution of the predominant bacteria at the genus level

Figure 4 revealed the relative abundance of bacterial phyla as a percentage in the two groups, with the top 7 predominant phyla being Firmicutes, Proteobacteria, Actinobacteria, Bacteroidetes, Fusobacteria, Thermi, and Verrucomicrobia. At the phylum level, the pre-treatment group was mainly composed of Proteobacteria (57.22%), Firmicutes (27.50%), Actinobacteria (7.40%), Bacteroidetes (6.41%) and Verrucomicrobia (1.47%), and the post-treatment group was mainly composed of Proteobacteria (60.47%), Firmicutes (23.12%), Actinobacteria (8.59%), and Bacteroidetes (7.31%) (Fig. 4A). At the genus level, the top 10 primary dominant genera are *Bifidobacterium*, *Streptococcus*, *Staphylococcus*, *Bacteroides*, *Prevotella*, *Enterococcus*, *Akkermansia*, *Lactobacillus*, *Phascolarctobacterium*, and *Haemophilus* (Fig. 4B).

### LEfSe analysis identified potential taxa associated with phototherapy

**Fig. 5:**
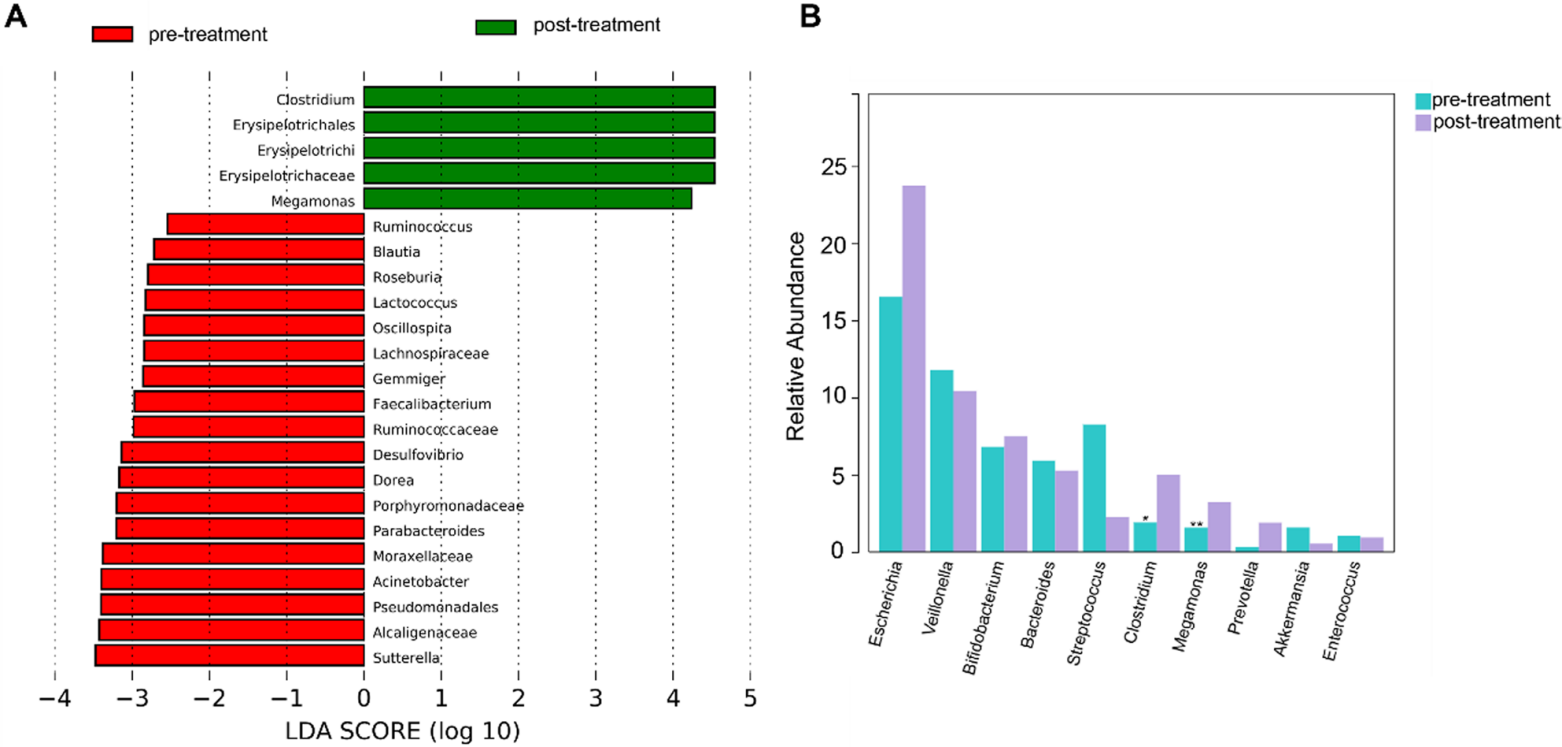
Differential gut microbiota identified by LEfSe analysis and corresponding abundance profiles. **A** LDA effect size (log10) plot showing the bacterial taxa with significant differences among groups (LDA score > 2.0). Each bar represents a taxon, with its length indicating the effect size. **B** Bar charts displaying the relative abundances of representative differential taxa identified by LEfSe in each group. (*P < 0.05, ** *P* < 0.01 vs. pre-treatment (before phototherapy) group; No mark of significance indicates *P* > 0.05). These results highlight distinct microbial signatures associated with blue light phototherapy

To identify microbial taxa significantly associated with phototherapy intervention, Linear Discriminant Analysis Effect Size (LEfSe) was performed using a Kruskal−Wallis test with α = 0.05 and a linear discriminant analysis (LDA) threshold of > 2.0. Taxa meeting both criteria (*p* < 0.05 and |LDA score| > 2.0) were considered significantly different. As shown in Fig. 5A, phototherapy induced significant alterations in intestinal microbiota composition. In the pre-treatment group, enriched taxa included members of the Porphyromonadaceae, Lachnospiraceae, Alcaligenaceae, Ruminococcaceae, and Moraxellaceae. In contrast, the post-treatment group exhibited enrichment of the class Erysipelotrichi, the order Erysipelotrichales, and the family Erysipelotrichaceae, indicating a compositional shift favoring Erysipelotrichi-related lineages. Genus-level comparisons in Fig. 4B further confirmed that the abundance of *Clostridium* (*P* = 0.026526) and *Megamonas* (*P* = 0.007653) was significantly increased. No species-level taxa passed the significance threshold (Supplementary Fig. S3). These findings indicate that blue light phototherapy is associated with specific alterations in gut microbial composition, potentially contributing to its physiological effects in neonates with hyperbilirubinemia.

### Gut microbial function analysis

To assess whether the observed microbial compositional shifts were accompanied by functional changes, predicted microbial functions were compared using PICRUSt2 based on COG, KEGG, and MetaCyc pathway databases. However, no statistically significant differences were detected between the post-treatment and pre-treatment groups across all databases (*P* > 0.05), at both higher-level functional categories and specific pathway levels (Supplementary Fig. S4). Despite the lack of statistically significant differences in functional pathways between groups, overall functional profiling revealed that the dominant microbial functions were broadly consistent across samples. The most abundant COG categories included carbohydrate transport and metabolism, amino acid transport and metabolism, and energy production and conversion. KEGG pathway analysis further showed that microbial genes were predominantly involved in core metabolic functions such as biosynthesis of ansamycins, biosynthesis of vancomycin group antibiotics, valine, leucine and isoleucine biosynthesis, and D-Alanine metabolism (Fig. 5A). Similarly, MetaCyc pathway profiles were enriched for pathways related to Cofactor, Prosthetic Group, Electron Carrier, and Vitamin Biosynthesis, Amino Acid Biosynthesis, Nucleoside and Nucleotide Biosynthesis, Fatty Acid and Lipid Biosynthesis, Carbohydrate Biosynthesis, and fermentation. These metabolic pathways are associated with liver function (Fig. 5B).

**Fig. 6:**
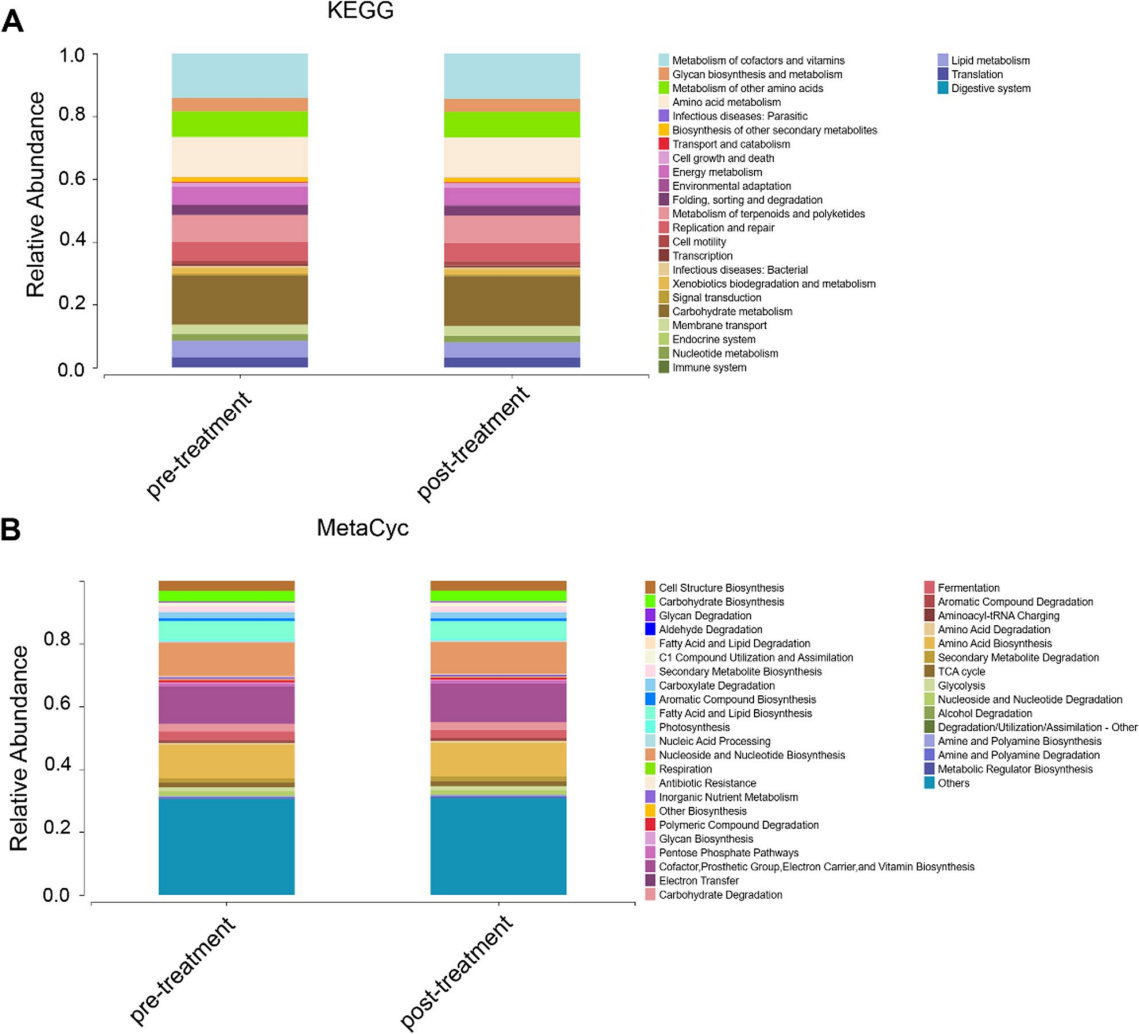
Gut microbiological function analysis. **A** Metabolic pathways using PICRUSt2 based on KEGG. **B** MetaCyc analysis based on PICRUSt2

## Discussion

Neonatal hyperbilirubinemia has a multifactorial etiology involving physiological processes, isoimmunization, genetic alterations, and environmental influences [13]. Emerging evidence implicates gut microbiota dysbiosis as a key environmental contributor, yet the underlying mechanisms linking microbiota to hyperbilirubinemia remain elusive [7, 14, 15]. Despite being the primary treatment for bilirubin reduction, phototherapy may inadvertently perturb this ecosystem [16, 17]. In this research, we compared the differences in gut microbiota in newborns with hyperbilirubinemia after receiving phototherapy. α diversity analysis showed that there were no differences in the total richness of gut microbiota between the two groups. This result revealed some agreement with the data that are currently published [18, 19]. Our data indicated a significant increase in the genera *Clostridium* and *Megamonas* and a marked community shift towards the class Erysipelotrichi. The clinical implications of these shifts warrant careful consideration. The significant β diversity differences (*P* < 0.001) and LEfSe-identified differentially abundant genera potentially related to phototherapy indicate that phototherapy disrupts microbial community structure. This aligns with reports linking blue light to reduced microbial diversity and altered taxa abundance [9, 19]. In comparison with the recent case-control study by You et al. [19], which highlighted Bacteroidetes as a biomarker enriched in neonates with pathologic jaundice compared to those with physiologic jaundice. This discrepancy likely stems from the distinct study designs: You et al. conducted a case-control comparison between disease states, whereas our self-controlled investigation focused on the specific perturbation induced by phototherapy within the same individuals. The divergent microbial signatures suggest that disease-associated dysbiosis and treatment-associated perturbations may operate through distinct ecological pathways. Pathological jaundice appears to be characterized by shifts at higher taxonomic levels, such as the phylum-level dominance of Bacteroidetes, whereas phototherapy produced reorganization primarily at the genus and species levels. Despite these compositional differences, both studies reported stable predicted microbial functional profiles, supporting the hypothesis of functional redundancy in the neonatal gut. Different taxa may preserve core metabolic capacities, including pathways related to the enterohepatic circulation of bilirubin. It should be noted, however, that the PICRUSt2-based results represent predicted rather than experimentally validated functions, and further multi-omics verification is required to confirm these findings.

Previous studies have indicated that *Bacteroides fragilis* of the phylum Bacteroidetes (*B. fragilis*) exerts pathogenic effects via tissue adhesion, immune evasion, and tissue destruction, accounting for 50% of neonatal bacteremia cases [20, 21]. Elevated *Bacteroides* abundance is further linked to chronic inflammatory diseases, including type 1 diabetes and celiac disease [22]. Our observations partially align with, yet importantly diverge from the case-control study by Akagawa et al. [18]. In their study comparing jaundiced neonates to healthy controls, Akagawa et al. identified a disease-associated dysbiosis characterized by a significant decrease in *Bifidobacterium*. They hypothesized that this reduction diminishes the suppressive effect on bacterial β-glucuronidase (β-GD) activity, potentially accelerating bilirubin deconjugation and enterohepatic circulation, thereby exacerbating hyperbilirubinemia. The stability of Bifidobacterium in our cohort post-phototherapy is a key differentiator. This might be attributed to our stringent exclusion of subjects with antibiotic or probiotic exposure, a key confounding factor that was not controlled for in the Akagawa et al. study, where some neonates received formula supplementation. Furthermore, while they reported an increase in *Enterococcus* in jaundiced neonates, our study revealed a different set of phototherapy-responsive genera (*Clostridium* and *Megamonas*). This suggests that phototherapy and the pathological state of jaundice itself may modulate the gut ecosystem through distinct mechanisms. The increase in *Clostridium*, a genus containing known β-GD producers, presents a compelling paradox that warrants further functional studies to elucidate the specific strains and metabolic pathways involved in the host response to phototherapy.

Yuan et al. [12]. investigated the adjunctive use of *Lactobacillus rhamnosus* GG during phototherapy in neonates with jaundice. Despite the small sample size and early termination of the trial, their findings suggested that probiotic supplementation alleviated phototherapy-associated dysbiosis by preserving α-diversity and accelerating post-treatment microbial recovery. In contrast, our study assessed phototherapy alone, revealing intrinsic microbial changes independent of probiotic buffering. The differences likely stem from intervention design, sample size, and follow-up duration, underscoring that co-interventions and study context strongly modulate microbial responses to phototherapy. Taken together, these comparisons indicate that observed microbiome changes are context-dependent, shaped by disease state, concurrent interventions, exclusion criteria, sampling timing, phototherapy parameters, and analytic pipelines.

## Limitations

This study indicates limitations. Firstly, the sample size was relatively small, and the absence of an independent healthy control group limits causal inference between phototherapy and microbial changes. Furthermore, the microbial signatures identified here were derived from a single-center, self-controlled study. To establish their generalizability and potential as clinical biomarkers, the associations between phototherapy and increased abundance of *Clostridium* and *Megamonas* require prospective validation in an independent, multi-center patient cohort. Secondly, the study relied on functional predictions via PICRUSt2 rather than direct experimental validation such as metagenomics, metatranscriptomics, or metabolomics. Thirdly, a key limitation of this study is the absence of an age-matched healthy control group without phototherapy exposure. The neonatal gut microbiota undergoes rapid and dynamic maturation during the first few weeks of life, and part of the observed compositional shifts may reflect natural temporal changes rather than phototherapy-specific effects. Furthermore, although we controlled for major confounders such as antibiotic and probiotic exposure and ensured breastfeeding, other factors, including maternal dietary factors and hospital environmental exposure, might still have influenced microbial profiles. Finally, this intestinal microbial testing only covered prokaryotic microorganisms and eukaryotic microorganisms, but did not include non-cellular microorganisms. Due to the scarcity of Internal Transcribed Spacer (ITS) sequences in eukaryotic microorganisms, library construction was unsuccessful. Thus, no analytical data were available. Future large-scale, multi-omics studies integrating experimental validation are needed to elucidate the causal mechanisms underlying the microbial regulation of bilirubin metabolism during phototherapy.

## Conclusion

In summary, our study provides comprehensive metagenomic evidence that blue light phototherapy significantly altered the gut microbial composition in NH, while preserving core metabolic functions. The observed shifts of *Clostridium* and *Megamonas* highlight the potential of these genera as potential taxa associated with phototherapy response for monitoring treatment response. Comparative analysis with related previous studies further reveals distinct and complementary microbial signatures associated with disease versus therapeutic intervention. These findings underscore the importance of considering both microbial composition and function in the management of NH. Future studies integrating multi-omics approaches and functional validation are needed to elucidate the mechanistic roles of intestinal microbes in bilirubin metabolism and to optimize microbiota-targeted therapies.

## Supplementary Information

Supplementary Material 1.

## Data Availability

The datasets generated during and/or analysed during the current study are available from the corresponding author on reasonable request.

## Abbreviations

NH: Neonatal hyperbilirubinemia
rRNA: Ribosomal RNA
LEfSe: Linear discriminant analysis effect size
OTUs: Operational Taxonomic Units
B. *fragilis*: *Bacteroides fragilis* of Bacteroidetes
β-GD: β-glucuronidase
ITS: Internal Transcribed Spacer
