# Supplementary material for: Impact of phototherapy on gut microbiota composition and function in neonates with hyperbilirubinemia: a metagenomic analysis
Source: BMC Pediatr. 2026 Feb 2;26:179. doi: 10.1186/s12887-026-06531-0 (PMC12955324; doi:10.1186/s12887-026-06531-0)
Supplement: Supplementary file 1 — Supplementary Material 1. [file 12887_2026_6531_MOESM1_ESM.docx]

**Supplementary materials for the main manuscript**


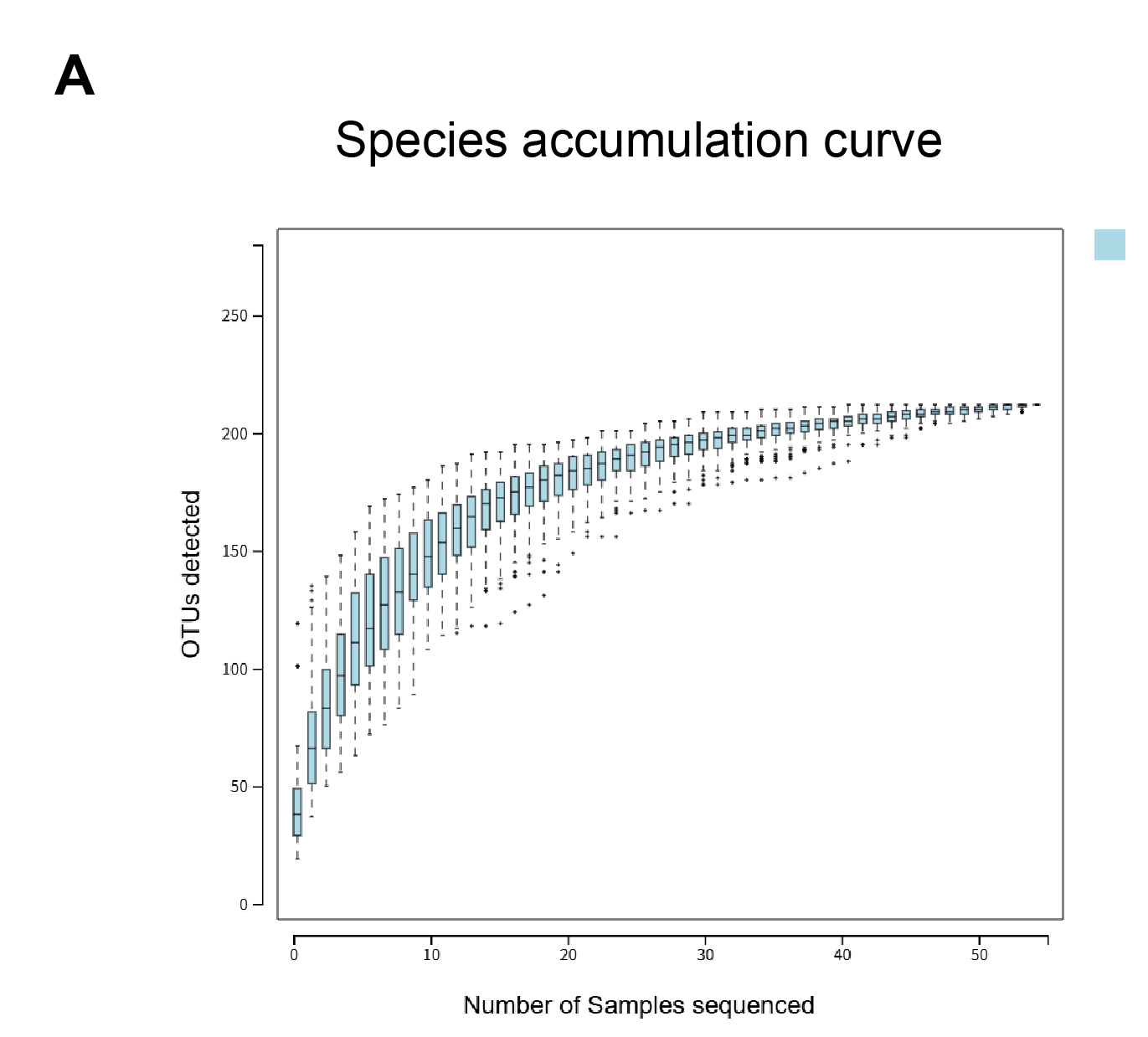


**Fig. S1** **Species accumulation curve assessing sampling adequacy for neonatal gut microbiota analysis.** The curve plots the cumulative number of observed Operational Taxonomic Units (OTUs) against the number of fecal samples sequenced. The plateau reached indicates that the sampling depth of n=52 samples (26 paired pre- and post-phototherapy specimens) was sufficient to capture the majority of microbial diversity present in the neonatal gut microbiota community under investigation.


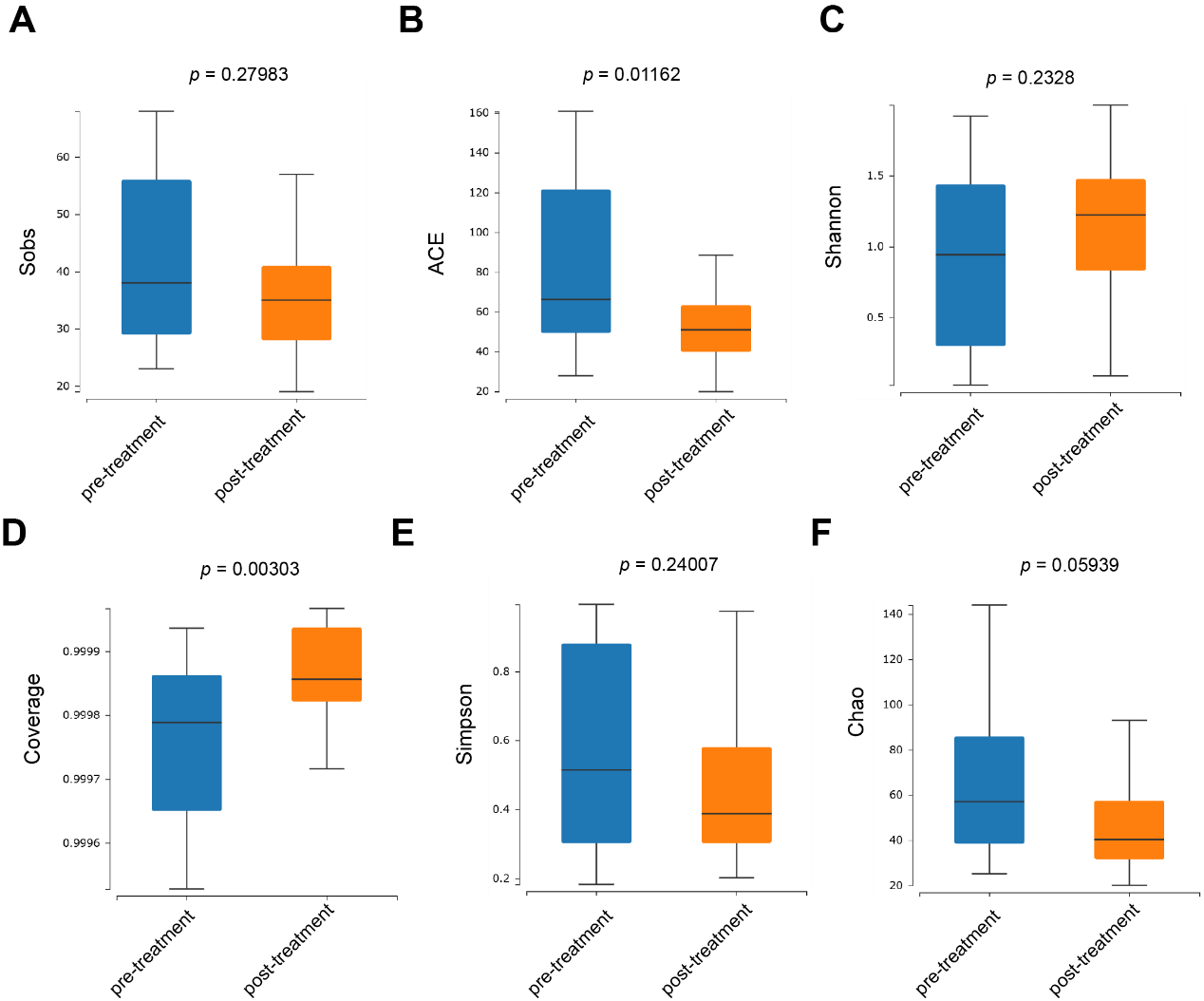


**Fig. S2.** Assessment of gut microbiota alpha diversity before and after phototherapy. Box plots show the within-sample diversity metrics for the pre-treatment and post-phototherapy groups. **A.** Sobs index, *P* = 0.27983; **B.** ACE index, *P* = 0.01162; **C.** Shannon index, *P* = 0.2328; **D.** Coverage index, *P* = 0.00303; **E.** Simpson index, *P* = 0.24007; **F.** Chao index, *P* = 0.05939. Significance levels between groups are indicated as follows: **P* < 0.05, ***P* < 0.01; absence of a mark indicates P > 0.05.


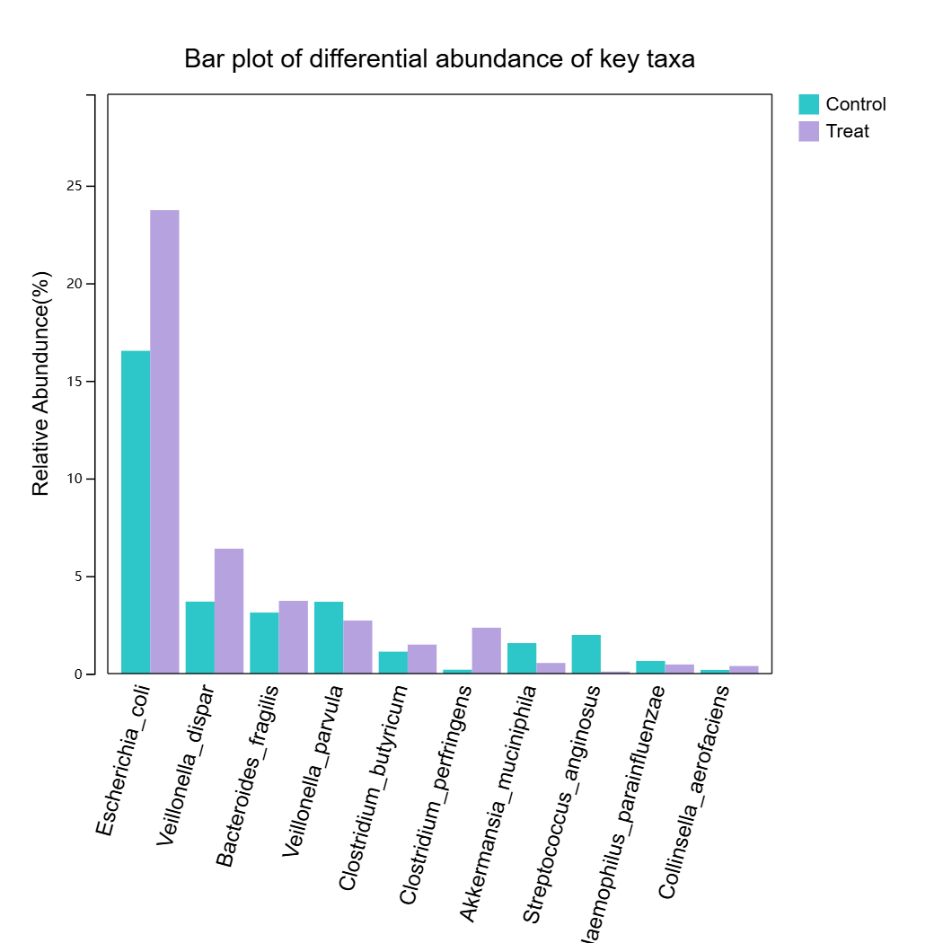


**Fig. S3.** Species-level relative abundance of significant differential taxa. Bar charts display the mean relative abundance of bacterial genera that were significantly different between the pre-treatment and post-phototherapy groups, as identified by Linear Discriminant Analysis Effect Size (LEfSe). Significance levels between groups are indicated as follows: **P* < 0.05, ***P* < 0.01; absence of a mark indicates P > 0.05.


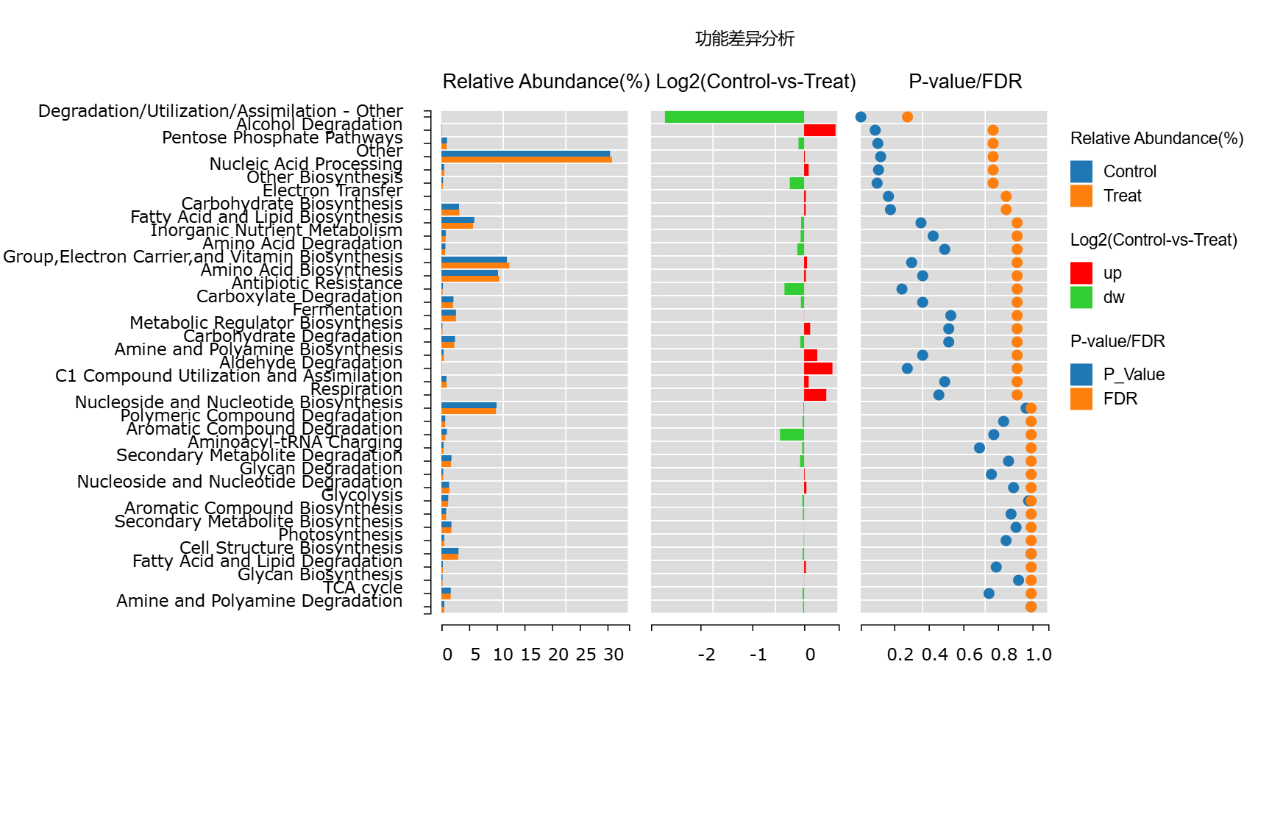


**Fig. S4.** MetaCyc analysis based on PICRUSt2. Box plots confirm no significant difference in key liver-related pathways (Wilcoxon *p* > 0.05).
